# Dyslipidemia among Pilots Visiting a Tertiary Care Centre: A Descriptive Cross-sectional Study

**DOI:** 10.31729/jnma.7968

**Published:** 2023-01-31

**Authors:** Bishwa Raj Dawadi, Khilasa Pokharel

**Affiliations:** 1 Department of Family Medicine, Grande International Hospital, Dhapashi, Kathmandu, Nepal; 2 Department of Microbiology, Kathmandu Medical College, Sinamangal, Kathmandu, Nepal

**Keywords:** *dyslipidemia*, *lipids*, *pilot*

## Abstract

**Introduction:**

Dyslipidemia is the imbalance of various lipids in our body like cholesterol, low-density lipoprotein cholesterol, triglycerides, and high-density lipoprotein. It has been established as a major factor associated with cardiovascular disease. The aim of our study was to find out the prevalence of dyslipidemia among pilots visiting a tertiary care centre.

**Methods:**

This descriptive cross-sectional study was conducted in the family medicine department of Grande International Hospital, Dhapasi, Kathmandu from 1 May 2022 to 30 July 2022 (Reference number: 08/2022). Seventy pilots were included in this study. Lipid profiles like total cholesterol, triglycerides, low-density lipoprotein cholesterol and high-density lipoprotein cholesterol were measured.

**Results:**

Among 70 pilots, only 2 (2.85%) (0-6.12, 90% Confidence Interval) had dyslipidemia with an increased level of triglycerides. Dyslipidemia was seen among pilots 41 to 60 years.

**Conclusions:**

Dyslipidemia among pilots was lower than in other studies done in similar settings.

## INTRODUCTION

Dyslipidemia is an imbalance of lipids and found to be associated with arthrosis and cardiovascular disease,^12^ that can cause incapacitation^3,4^ and disability.^5^

Dyslipidemia is one of the risk factors for various cardiovascular diseases.^6^ Assessment of dyslipidemia in youth is important to design the primary preventive program. From the previous study, it has been reported that dyslipidemia is associated mainly with males, poor glycemic control and hypertension. Age, smoking, and obesity are other predisposing factors for dyslipidemia.^7^ Regular monitoring of blood glucose, lipid profile and other changes in the lifestyle such as weight loss, physical exercise, and proper medication is suggestive.

The aim of the study was to find out the prevalence of dyslipidemia among pilots visiting a tertiary care centre.

## METHODS

This descriptive cross-sectional study was conducted in the family medicine department of Grande International Hospital, Dhapasi, Kathmandu from 1 May 2022 to 30 July 2022. Ethical approval was obtained from Institutional Review Committee (Reference number: 08/2022). Convenience sampling was used. A proforma was created following which data was collected. The inclusion criteria of this study include pilots who are above 18 years and provided written and informed consent. Exclusion criteria include pilots who are not able to communicate via verbal or non-verbal clues, who are in delirium, who are taking antidepressant before enrollment in the study and the one who does not want to participate in the study. The sample size was calculated using the following formula:


$$\begin{array}{ll} \text{n} & ={\text{Z}}^{2}\times \frac{\text{p}\times \text{q}}{{\text{e}}^{\text{2}}}\\ & =1.645^{2}\times \frac{0.50\times 0.50}{0.1^{2}}\\ & =68 \end{array}$$

Where,

n = minimum required sample size
Z = 1.645 at 90% Confidence Interval (CI)
p = prevalence taken as 50% for maximum sample size
q = 1-p
e = margin of error, 10%

The calculated sample size was 68. However, we took 70 samples.

General physical examination includes clinical presentation like headache, dizziness, shortness of breath, fatigue, sleep problem and palpitation. The laboratory investigations that were conducted included total cholesterol, triglycerides, high-density lipoprotein cholesterol and low-density lipoprotein cholesterol. The cut-off value for the lipid profile indicated was defined according to the standard manual.^8^ The adverse lipid concentration were defined as follows: High-density lipid-Cholesterol >35 mg/dl, low-density lipid-Cholesterol 1-130 mg/dl, triglycerides <150 mg/ dl and total cholesterol <240 mg/dl. Dyslipidemia was defined as an adverse concentration in one or more of the four lipids.

Data were analyzed using Microsoft Excel 2016 and IBM SPSS statistics version 18.0.

## RESULTS

Among 70 pilots, 2 (2.85%) (0-6.12, 90% Confidence Interval) had dyslipidemia with an increased level of triglycerides. Dyslipidemia was seen among the pilots aged 41 to 60 years.

## DISCUSSION

The present study described the presence of adverse serum lipid in 2022 among pilots of Nepal. During these three months study high density lipid cholesterol, low density lipid cholesterol, triglycerides and total cholesterol profiles were observed. In this study only 2.85% of the pilots included were found to be having dyslipidemia. Similar type of study was conducted where 33.1% pilots^9^ had dyslipidemia which is more than our study but was below the average margin. When similar type of study was conducted by other group higher prevalence 61.7% of pilots^10^ had dyslipidemia. These inconsistent results may be due to diet factors and obesity. In our study, dyslipidemia is diagnosed among the pilots of age group between 41 to 60 years, whereas in other age group between 18 to 40 years dyslipidemia was not diagnosed. Similar type of study was followed which review changes of lipids that occurs in men and women during aging and potential mechanism of age related disorders of lipoprotein.^11^ This could be because there is a significant interaction between age and education level on dyslipidemia. In this study, among total pilots included 94.28% were male and 5.71% were female. There are many attribute which shows men's domination of aviation sector to Nepal's patriarchal society and deeply ingrained gender role. It's been a national culture which encourage women less adventurous jobs.^12^ There are several limitations in the present study that need to be considered. Limitation of the study includes study only target small number of pilots of Nepal, number of pilots included in the current study was less than that of large scale survey, the study period was short and since the target population were not willing to give consent for survey of other parameters, so other parameters were not included in the study.

## CONCLUSIONS

Dyslipidemia among pilots was lower than in other studies done in similar settings. The common dyslipidemia was triglycerides. The reason for these trend is unknown so it should be further explored.
